# Therapeutic pathways for ischemic stroke patients in France: a national hospitalization database study

**DOI:** 10.3389/fneur.2026.1741750

**Published:** 2026-05-19

**Authors:** Laurent Derex, Jean Tardu, Didier Caumette, Julien Chollet, Elise Cabout, Charlotte Cancalon, Françoise Bugnard, Agathe Lollichon, Didier Smadja

**Affiliations:** 1 Stroke Center, Neurology Department, Lyon Neurological Hospital, RESHAPE, Inserm U1290, University of Lyon, Lyon, France; 2 Boehringer Ingelheim France, Paris, France; 3 stève consultants, Oullins-Pierre-Bénite, France; 4 CH Narbonne, Narbonne, France; 5 Stroke Unit, INSERM U-1266 and U895, Department of Neurology, Centre Hospitalier Sud-Francilien, Paris-Saclay University, Corbeil-Essonnes, France

**Keywords:** ischemic stroke, management, mechanical thrombectomy, non-interventional study, real-world evidence, therapeutic pathways, thrombolysis

## Abstract

**Introduction:**

Ischemic strokes are of major public health impact in France, with more than 120,000 hospitalizations each year. Its main management relies on the possibility to perform thrombolysis within the first hours following the stroke in emergency room, stroke unit (SU) or with telemedicine. The objective of this study was to establish an overview of hospital care of stroke and identify therapeutic pathways, and their characteristics in France overall and by region.

**Methods:**

This is a non-interventional descriptive cross-sectional study using secondary data from the French national hospitalization database (PMSI). Hospital stays presenting a diagnosis of ischemic stroke between January 1, 2022, and December 31, 2023, were selected. Among them, therapeutic pathways were identified based on the type of care facility, the presence of mechanical thrombectomy, inter-hospital transfer, and type of discharge.

**Results:**

In 2023, 171,674 patients were hospitalized for stroke with 57.4% for ischemic stroke. At national level, 70% of hospital stays for ischemic stroke in 2023 were managed in SU, whereas these facilities represent only 13% of facilities in France. The network of SU providing stroke care is highly dependent on the region. Among all hospital stays, 78 distinct therapeutic pathways of management of ischemic stroke were identified. Among them, 11 represented 90% of the study population.

**Conclusion:**

The territorial organization of establishments, particularly those with a SU, is a key factor in the management of ischemic stroke. This optimization of pathways can have an impact on costs, length of stay, and the probability of returning home.

## Introduction

In France in 2022, more than 120,000 individuals were hospitalized for stroke, and more than 30,000 died from a stroke (1). In addition to its high mortality rate, stroke sequelae are one of major causes of acquired non-traumatic motor disability and dementia. Due to population aging, the number of strokes is expected to increase, with 7.8 million stroke-related deaths expected worldwide by 2030 (2). From a local economic point of view, the burden of stroke is also heavy, with nearly 3.5 billion euros reimbursed by the Health Insurance in France (3).

The 2010–2014 national stroke action plan defined and structured the French territorial network of stroke management (4). This plan aimed to structure the territorial organization for a rapid and high-quality care for any suspected stroke.

According to this national plan, stroke management typically begins with a call to emergency medical services (SAMU), which are in charge of referring patients to neighboring hospitals, notably based on location, criticality and infrastructures available. Ideally, patients are referred to facilities with stroke expertise, which can provide a positive diagnosis (usually by magnetic resonance imaging–MRI or computed tomography–CT) and appropriate management, on a 24/7 basis (5). The main therapeutic procedure is the intravenous thrombolysis with rt-PA, which must be performed within 4 h 30 min after stroke onset. Thrombolysis is prescribed by a neurologist and monitored through the whole administration. Finally, following thrombolysis, and in the presence of proximal artery occlusion, mechanical thrombectomy (MT) may be performed in specialized centers. Endovascular therapy is recommended up to 24 h after the onset of symptoms (6, 7). In France, stroke expertise is recognized by regional health agencies, which grant authorizations based on the type of facility and the type of procedures performed in the hospital. Three levels of expertise exist. The first level is for hospitals where only thrombolysis can be done. The next two levels gather facilities where both thrombolysis and thrombectomy can be done with a different level of thrombectomy capacity. Level three centers also have neurointensive care and 24/7 on-site critical care coverage. For international transposability purposes, facilities with stroke expertise were considered as stroke units (SU). Among SU, thrombectomy-capable stroke centers (TSC) included centers performing at least 25 TM per year. Conversely, other SUs corresponded to non-TSCs stroke facilities.

If no SU is available within a short period (i.e, within primary care facilities–PCF), patients are referred to emergency rooms (ER), and telestroke can be used to optimize decision-making. Telestroke consists of a medical examination via secure data transfer services and videoconferencing between stroke physicians and on-site assistance from an emergency physician. Thrombolysis is co-prescribed by the on-call neurologist carrying out the telestroke procedure and administered before the patient transfer to a SU.

The positive impact of SU and telestroke systems was described in the 2017 report from the French National Health Authority (Haute Autorité de Santé). In this study, among the 550 participating healthcare facilities, 139 had SU and 200 had telestroke. Among centers with telestroke, the percentage of neurovascular consultations and post-stroke follow-up was higher, and imaging delays were reduced (8–10). Thus, telestroke should allow (1) homogeneous coverage of the territory in terms of access to care, (2) more effective stroke diagnosis and selection of appropriate care, and (3) reduction of patient transfer times and faster care.

The management of stroke is based on the territorial organization of facilities with SU or telestroke, and the potential access to thrombolysis and thrombectomy in these facilities. The availability of tools and treatments for quicker and easier management of ischemic strokes would save a considerable amount of time and resources in the context of organizational challenges in a disease for which time is of the utmost importance.

Therefore, this study aimed to have an overview of hospital care of stroke in 2022 and 2023 in France, and to identify hospital care pathways, and their characteristics, at a national and regional level.

## Methods

### Design

This was a non-interventional descriptive study based on cross-sectional study using secondary data from the French national hospitalization database (PMSI)–using data from medical, surgical and obstetrics care (*medicine, chirurgie, obstétrique*–PMSI-MCO). PMSI database contains all hospital stays data and related claims irrespective of the healthcare insurance system and setting (public/private) and it is exhaustive in terms of stays, procedures, and management costs. Data available in PMSI-MCO includes patients characteristics (e.g., age, sex) and information on hospitalization (e.g., primary and secondary diagnoses, medical units, expensive treatments, admission origin and discharge destination, main procedures undergone, hospitalization costs). This study was carried out in compliance with French regulations on access to the PMSI database through MR-006 process.

### Patient selection

The study population (Stroke population) was defined as all hospital stays occurring between January 1st, 2022 and December 31st, 2023, with either a primary or a secondary diagnosis of stroke-related ICD-10 code (International Classification of Diseases, 10th Revision) [I60, I61, I62, I63, I64, or G45 (Table 1)] and an admission from ER, home or medical-social accommodation facilities.

**Table 1:** Stroke-related ICD-10 code.

ICD-10 code	Label
I60	Subarachnoid hemorrhage
I61	Intracerebral hemorrhage
I62	Other nontraumatic intracranial hemorrhage
I63	Cerebral infarction
I64	Stroke, not specified as hemorrhage or infarction
G45	Transient cerebral ischemic attacks and related syndromes

For assessing therapeutic pathways, a sub-population (Ischemic Stroke population), including only hospital stays occurring in 2023 (admission and discharge dates in 2023), with a primary diagnosis of ischemic stroke (I63) was defined and considered as index hospitalizations of therapeutic pathways.

### Outcomes and analyses

The overview of strokes in France was described among the Stroke population. The numbers of hospitalizations and hospitalized patients were assessed overall and by type of stroke, while the main socio-demographic characteristics (i.e., age and sex) were described at admission. Incidence rates were estimated per 10,000 French individuals, by using the number of individuals in France per region in 2022 and 2023 using data from the French national census (INSEE).

Therapeutic pathways were identified within the Ischemic Stroke population, based on the type of facility of index hospitalization (TSCs, other SU, PCF), the type of MT procedure done, the presence of inter-hospital transfers, and the final discharge modality. For this analysis, ER admission, MT, post-MCO destinations, cumulative length of stay and cumulative costs were described on Ischemic Stroke population. SU were identified based on the available list of such facilities. TSCs were identified as SU with at least 25 thrombectomy stays in 2023. Five post-MCO destinations were considered: (1) home, (2) post-acute care and rehabilitation (SSR), (3) home care (HAD), (4) short stay hospital ward (USCD), psychiatry (PSY), long stay hospital ward (USLD), and (5) death. Cumulative length of stays and cumulative costs were calculated by summing length of stays and costs of all hospitalizations included in individual pathways. Region was defined as the region of the facility of index hospitalization.

Direct costs were described from a hospital perspective, based on the French national methodology (*Etude nationale des coûts à méthodologie commune*–ENC), as well as a payer perspective, using reimbursed tariffs (DRG) and supplements (specific wards, expensive drugs, and medical devices…).

Continuous variables were summarized by mean, median, standard deviation (SD), first and third quartile, number of missing data, minimum, and maximum. Categorial variables were described by absolute and relative frequency of each category. Analyses were conducted using SAS (version 9.4 or later) statistical software (SAS Institute Inc., Cary, NC, US).

## Results

### Overview of stroke in France

#### Number of hospitalized patients and characteristics

In 2022, 176,368 patients were hospitalized for stroke, corresponding to 198,648 hospital stays (Table 2). Based on hospital diagnosis codes, half (56.4%) of stays were for ischemic stroke, 22.0% for hemorrhagic stroke, 21.3% for transient ischemic stroke and 5.4% for unspecified strokes.

**Table 2:** Description of hospital stays for stroke in 2022 and 2023.

Characteristics	2022	2023
Number of patients	176,368	171,674
Ischemic stroke	102,014 (57.8%)	98,624 (57.4%)
Hemorrhagic stroke	38,875 (22.0%)	38,369 (22.3%)
Transient stroke	39,805 (22.6%)	38,948 (22.7%)
Unspecified stroke	9,693 (5.5%)	8,963 (5.2%)
% males	92,813 (52.6%)	90,477 (52.7%)
Sex-ratio M/F	1.11	1.11
Age–mean (sd)	72.3 (15.9)	72.1 (15.8)
Number of hospital stays	198,648	193,190
Ischemic stroke	112,038 (56.4%)	108,394 (56.1%)
Hemorrhagic stroke	43,738 (22.0%)	43,106 (22.3%)
Transient stroke	42,365 (21.3%)	41,331 (21.4%)
Unspecified stroke	10,752 (5.4%)	10,012 (5.2%)
% males	105,617 (53.2%)	103,026 (53.3%)

In 2023, the number of hospital stays for stroke decreased by 2.7% (193,190 hospital stays) compared with 2022 and similar distribution across stroke types was observed.

The mean (SD) age at admission was 72.3 (15.9) years in 2022 and 72.1 (15.8) years in 2023. The sex ratio for hospital stays was 1.14 corresponding to 53.3% of males without any significant variation between 2022 and 2023.

No significant differences are observed between 2022 and 2023. Therefore, only the year 2023 is retained for further analysis to highlight the most recent results and ensure clarity and clinical relevance.

The incidence rate of stroke hospitalizations in metropolitan France was 28.33 per 10,000 French individuals in 2023 (Table 3), with higher rates in Bretagne (36.18) and in Nouvelle-Aquitaine (34.72), and lower rates in Ile-de-France (24.30) and in Grand-Est (25.52) regions.

**Table 3:** Incidence rates by region of stroke and ischemic stroke in 2023.

French regions	Stroke incidence (per 10,000)	Ischemic stroke incidence (per 10,000)
Auvergne-Rhône-Alpes	25.95	13.96
Bourgogne-Franche-Comté	31.58	18.26
Bretagne	36.18	20.07
Centre-Val de Loire	25.69	14.08
Corse	29.58	15.40
Grand Est	25.52	14.92
Hauts-de-France	26.37	15.37
Île-de-France	24.30	13.12
Normandie	30.12	17.76
Nouvelle-Aquitaine	34.72	19.69
Occitanie	29.36	17.26
Pays de la Loire	27.53	15.14
Provence-Alpes-Côte d’Azur	31.95	16.90
Metropolitan France	28.33	15.85

In terms of ischemic stroke, the hospitalizations incidence rate was 15.85 per 10,000 French individuals, with the same variations between regions observed for overall stroke.

#### Stroke management

At the national level, 69.5% of hospital stays for ischemic stroke in 2023 were managed in SU facilities (including TSCs), whereas these facilities represented only 12.9% of facilities in France.

The network of SU facilities providing stroke care is highly dependent on the region. Indeed, the Hauts-de-France region had 20% of SU, while the Provence-Alpes-Côte d’Azur and Pays de la Loire regions had only 8% of SU (Table 4).

**Table 4:** Distribution of facilities with SU and distribution of ischemic strokes managed in SU by region.

French regions	Number of facilities			Number of hospital stays for ischemic stroke	
	With SU	Without SU (i.e., primary care facility–PCF)	Total number of facilities	In SU	In PCF
Auvergne-Rhône-Alpes	18 (11.8%)	134 (88.2%)	152 (12.7%)	7,400 (64.7%)	4,044 (35.3%)
Bourgogne-Franche-Comté	7 (11.5%)	54 (88.5%)	61 (5.1%)	2,955 (58.1%)	2,133 (41.9%)
Bretagne	8 (13.1%)	53 (86.9%)	61 (5.1%)	5,098 (74.0%)	1,787 (26.0%)
Centre-Val de Loire	6 (13.3%)	39 (86.7%)	45 (3.8%)	2,299 (63.5%)	1,322 (36.5%)
Corse	1 (12.5%)	7 (87.5%)	8 (0.7%)	233 (43.1%)	308 (56.9%)
Grand-Est	15 (13.0%)	100 (87.0%)	115 (9.6%)	5,837 (70.3%)	2,463 (29.7%)
Hauts-De-France	17 (20.0%)	72 (80.0%)	90 (7.5%)	7,494 (81.5%)	1,698 (18.5%)
Ile-de-France	21 (12.5%)	147 (87.5%)	168 (14.1%)	11,761 (72.5%)	4,459 (27.5%)
Normandie	8 (13.3%)	52 (86.7%)	60 (5.0%)	4,171 (70.8%)	1,719 (29.2%)
Nouvelle-Aquitaine	17 (13.9%)	105 (86.1%)	122 (10.2%)	8,635 (71.8%)	3,397 (29.2%)
Occitanie	18 (15.5%)	98 (84.5%)	116 (9.7%)	8,761 (83.2%)	1,771 (16.8%)
Pays-de-la-Loire	5 (8.3%)	55 (91.7%)	60 (5.0%)	3,235 (54.7%)	2,679 (45.3%)
Provence-Alpes-Côte d’Azur	8 (8.2%)	90 (91.8%)	98 (8.2%)	4,893 (56.1%)	3,825 (43.9%)
DROM	4 (10.5%)	34 (89.5%)	38 (3.2%)	2,519 (62.8%)	1,492 (37.2%)
Total	154 (12.9%)	1,040 (87.1%)	1,194 (100.0%)	75,300 (69.5%)	33,101 (30.5%)

SU, stroke unit; TSC, thrombectomy-capable stroke center; PCF, primary care facility.

Therefore, the proportion of hospital stays for ischemic strokes managed in SUs varied over region, with 82% in Hauts-de-France, and 55% in Pays de la Loire.

### Therapeutic pathways

#### Characteristics

A total of 65,808 index hospital stays were identified in 2023, which constituted the Ischemic Stroke population. Overall, 78 distinct therapeutic pathways were identified (Supplementary materials, Supplementary Table 1).

Among the 65,808 ischemic stroke pathways, around 35% were directly admitted in a TSC, 38% in another SU and 27% in a PCF. Along the whole care pathway, around 38% of pathways ultimately involved a passage through a TSC. Around 5% of therapeutic pathways included MT procedures and 9% had at least one inter-hospital transfer, 29% of which were to an SU (Table 5). For the final discharge modality, 64.8% were home, 22.3% post-acute care and rehabilitation and 9.3% death. Finally, stroke pathways were almost exclusively initiated in public hospitals, with only 1,678 (2.5%) pathways in private settings.

**Table 5:** Characteristics of pathways within the Ischemic stroke population.

Characteristics	Pathways (*N* = 65,808)
	*n* (%)
Type of facility at ischemic stroke admission (index hospital stay)	
TSC	23,290 (35.4%)
Other SU	24,814 (37.7%)
PCF	17,704 (26.9%)
Passage through a TSC along the ischemic stroke pathway	
No	41,099 (62.5%)
Yes	24,709 (37.6%)
Thrombectomy procedure along the pathway	
No MT	62,283 (94.6%)
One or more MT	3,525 (5.4%)
At least one inter-hospital transfer	
No	59,835 (90.9%)
Yes	5,973 (9.1%)
Discharge	
Discharge 1: Home	42,653 (64.8%)
Discharge 2: SSR	14,696 (22.3%)
Discharge 3: HAD	1,549 (2.4%)
Discharge 4: USCD, PSY, USLD	794 (1.2%)
Discharge 5: Death	6,116 (9.3%)
Cumulative length of stay in overnights	
Mean (sd)	10.1 (10.7)
Median (Q1–Q3)	7.0 (4.0–13.0)
Min–Max	0.0–241.0
Total cumulative cost– € (missing = 520)	
Mean (sd)	8,132.5 (5538.9)
Median (Q1–Q3)	6,024.9 (4159.6–9451.3)
Min–Max	610.1–107010.86

SU, stroke unit, TSC, thrombectomy-capable center, PCF, primary care facility, MT, mechanical thrombectomy, SSR, post-acute care and rehabilitation; HA, home care; USCD, short stay hospital ward; PSY, psychiatry; USLD, long stay hospital ward.

The mean (SD) cumulative length of stay was 10.1 (10.7) overnights, and the mean (SD) cumulative cost of therapeutic pathways was €8,132.50 (5538.90).

#### Main therapeutic pathways

Among the 78 therapeutic pathways, 11 pathways represented 90% of the ischemic stroke population (Table 6).

**Table 6:** Therapeutic pathways most represented among the study population in 2023.

Therapeutic pathways	Number of pathways	Length of pathways (overnights)	Total ENC costs (€)
	(*N* = 65,808)		
Other SU—No MT—discharge 1	16,108 (24%)	7.3	6,251
TSC—No MT—discharge 1	13,889 (21%)	6.7	6,086
PCF—No MT—discharge 1	9,293 (14%)	8.0	6,208
Other SU—No MT—discharge 2	5,111 (8%)	16.4	9,451
TSC—No MT—discharge 2	3,819 (6%)	15.2	9,457
PCF—No MT—discharge 2	3,351 (5%)	15.7	9,041
Other SU—No MT—discharge 5	1,996 (3%)	11.3	10,244
PCF—No MT—discharge 5	1855 (3%)	10.84	9,672
TSC—No MT—discharge 5	1,308 (2%)	10.4	10,628
TSC—MT—discharge 1	988 (2%)	11.2	15,711
TSC—MT—discharge 2	905 (1%)	18.5	18,993

SU, stroke unit, SC, stroke center, TSC, thrombectomy stroke center, MT, mechanical thrombectomy, PCF, primary care facility, discharge 1, home, discharge 2, SSR, discharge 3, HAD, discharge 4, USCD, PSY, USLD, discharge 5, death.

The most common therapeutic pathways were those managed in SUs and those whose discharge modality was home. None of these pathways involved inter-hospital transfers.

Therapeutic pathways with a discharge at home were associated with shorter length of stays and lower costs, while pathways including MT had longer length of stays and higher total costs.

At a regional level, pathways starting in a SU, without thrombectomy and with discharge at home were also the most frequent, except in Ile-De-France, where the most frequent pathways were the ones starting in a TSC.

**Figure 1:**
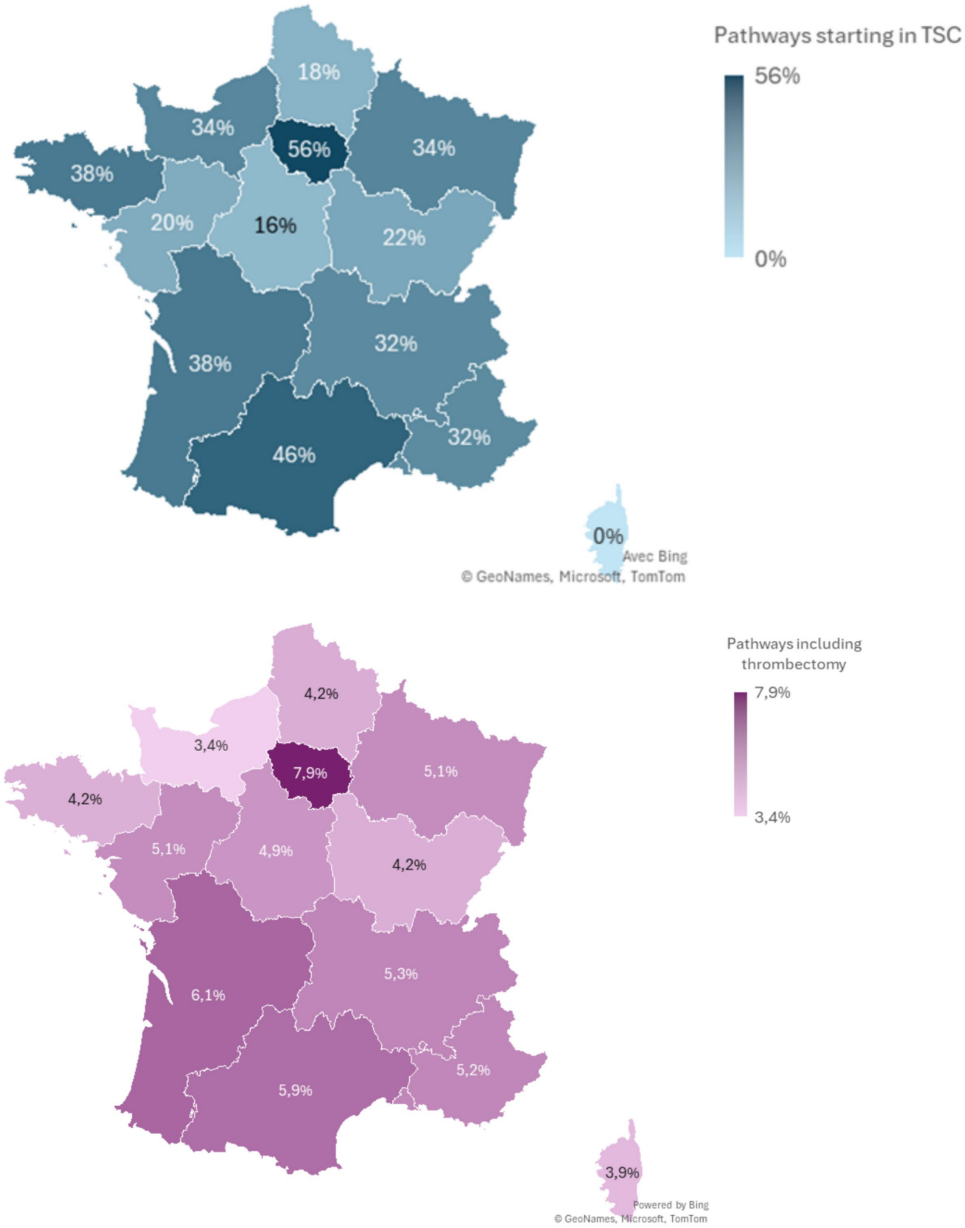
Proportion of pathways starting in TSC and proportion of pathways including thrombectomy.

The proportion of pathways starting in TSC and the proportion of pathways with MT at a regional level were displayed in Figure 1.

## Discussion

Ischemic stroke is a major public health issue in France. Thanks to the national stroke action plan, a highly interconnected regional network with optimized territorial organization between facilities with stroke expertise and with or without neurosurgery and interventional neuroradiology structured a care network for suspected strokes in coordination with a SU (telestroke).

This study conducted on PMSI data provide an overview of the current management of stroke in France, with a focus on ischemic stroke. In particular, this study showed the impact of the national stroke action plan on the deployment of SUs, with a majority of patients admitted to an SU at index stays. This tends to optimize patients’ management with the most specialized healthcare units, from the diagnosis to the need for further therapies such as thrombolysis and/or mechanical thrombectomy.

In 2023, 193,190 hospital stays were identified with a stroke diagnosis, of which 56.1% were ischemic strokes. No change was observed between 2022 and 2023 in type of stroke. In 2023, adult men and women suffered strokes in equal numbers, with similar mean ages of around 71.8 years old. The repartition of types of strokes (ischemic, hemorrhagic, transient or unspecified) did not change with age or gender. These findings are not entirely consistent with the literature (1, 11, 12) as Olié et al. (12) and Gabet et al. (1) showed a rate of 78.0% of ischemic stroke. Of note, the definition of the study population was different as we considered both primary and secondary diagnosis of stroke for enrolment in the study, whereas Olié et al. only considered patients with a primary diagnosis. Furthermore, we only considered diagnosis of ischemic stroke (I63), whereas the Olié et al. (12) defined ischemic stroke population by mechanical thrombectomy or code I63 or I64.

Overall, 69.5% of hospital stays for ischemic stroke in 2023 were managed in SUs, whereas these facilities represented only 12.9% of facilities in France, highlighting the importance of these centers in stroke management. The increase in the number of SUs between 2013 and 2023 has increased the proportion of strokes managed in SUs. At a regional level, some differences were observed depending on how each regional stroke network is organized. Some regions had a strong network of SUs, while others had fewer. In these regions, ischemic stroke management was likely based on a strong network of facilities using telestroke.

This study of therapeutic pathways has provided a full picture of ischemic stroke hospital management in France. In total, 90% of the patients diagnosed with an ischemic stroke could be gathered in 11 main therapeutic pathways, and few inter-hospital transfers were observed. Results highlighted that 5% of hospital stays included mechanical thrombectomy and that the most common discharge modalities were home (65% of hospital stays), and post-acute care and rehabilitation (22% of hospital stays). In addition, the most frequent therapeutic pathways were those managed by an SU for index hospitalization.

In 2014, the number of SUs was 135 according to the article by Com-Ruelle et al. (13). In 2023, our study identified 154 SUs, representing an increase of 14%. Com-Ruelle et al. showed that 61% of patients were hospitalized in a SU. The increase observed between 2010 and 2014 in Com-Ruelle et al. has been confirmed with our finding of 73.1% in 2023. Similar regional disparities were already observed in 2014. The identification of SUs was based on a published list, which seems consistent with the number of facilities identified in 2014 by Com-Ruelle et al. (13) and the deployment of these facilities. The identification of TSC in PMSI data was based on the expert opinion of at least 25 stays related to a MT in 2023 but the number of TSC identified was consistent with what is known about stroke management in France and was confirmed by experts.

The article by Com-Ruelle et al. (13) showed that half of patients with a stroke returned home or to an institution after their first hospital stay. Only 27% of patients were referred to and hospitalized in a follow-up and rehabilitation care unit. Our results showed an evolution between 2012 and 2023 in discharge modality with an increase in the proportion of patients returning home (64.8%). This increase is consistent with the rise in the number of SUs. Indeed, even if changes in practices may have occurred over 10 years, our results suggested that patients managed in an SU were more likely to return home.

According to the latest report of French Cour des Comptes on stroke management in France, around 54% of ischemic strokes are managed in SUs, which is notably lower than the 69.5% observed in our study (14). This report is based on studies accounting the I64 code as a potential ischemic stroke, which could explain part of this difference, as these unspecified strokes are usually not managed in SUs. Nonetheless, I64 code only accounted for 5% of strokes in our study, limiting the impact of this selection bias. Another reason explaining this difference, could be the number of hospitals labeled as SU. The Cour des Comptes report identified 141 SUs, while our study considered 154 centers as mentioned above, directly impacting the proportion of strokes managed in such units/centers.

In terms of costs, the article by Schmidt et al. (15) reported the cost of stays for the acute phase for patient with ischemic stroke treated in SU/SC of €6,199.81. Our findings were consistent at €8,132.5 per patient, considering changes in practices and the increase in costs that may have occurred over 10 years.

The study was a non-interventional study using secondary data from the PMSI. The use of the PMSI database allowed capturing data on all patients hospitalized for a stroke in France and provided a unique opportunity to get exhaustive information on therapeutic pathways, reimbursed healthcare consumptions, procedures, and costs. However, there may be some limitations related to homogeneity of coding procedures. Real-word data can be limited by misclassification, an under- or mis- reporting of diagnoses (thrombectomy procedures, inter-hospital transfers, stays of less than 24 h for thrombectomy, or emergency room visits to a primary receiving facility prior to transfer to a SU for stroke management) resulting in a risk of information bias, even if limited (16, 17). In addition, estimates of incidence of ischemic stroke based on the French PMSI should be used with caution due to the high proportion of false-negative cases (32.7% of ischemic stroke), leading to an underestimation of the incidence of ischemic stroke (18).

## Conclusion

The current burden of ischemic stroke in France is high. Its management begins with confirmation of the case in an emergency department or directly in a facility with a SU. Thrombolysis must be performed within the first few hours following the stroke. The management of ischemic stroke depends on the local presence of facilities with SU, and specifically with interventional neuroradiology, as well as the deployment of a telemedicine network specialized in stroke counseling (telestroke).

Despite the territorial disparities, the management of ischemic stroke can be summarized in 11 synthetic pathways. The most frequent therapeutic pathways were those involving management in a SU, without inter-hospital transfer, without thrombectomy and with a discharge to home. In regions with the fewest SUs, the management of ischemic stroke is likely based on the use of telestroke. In this case, inter-hospital transfers were more frequent for thrombectomy or more optimal management in an SU.

Optimizing stroke management with the presence of SUs throughout France and the deployment of telemedicine, may have an impact on the length of the treatment pathway, the associated cost and patient outcome.

## Data Availability

Access to SNDS data is restricted to qualified and authorized personnel only. No individual data can be extracted from the secured platform, as per French national data protection agency guidelines, further inquiries can be directed to the JT, jean.tardu@boehringer-ingelheim.com.
