# Supplementary material for: Therapeutic pathways for ischemic stroke patients in France: a national hospitalization database study
Source: Front Neurol. 2026 May 19;17:1741750. doi: 10.3389/fneur.2026.1741750 (PMC13228163; doi:10.3389/fneur.2026.1741750)
Supplement: Supplementary file 1 [file Table_1.pdf]

Supplementary material

The online version contains supplementary material

Table 1. Description of the 78 identified therapeutic pathways

Number	Therapeutic pathways
1	TSC ---- MT ---- discharge 1
2	TSC ---- MT ---- discharge 2
3	TSC ---- MT ---- discharge 3
4	TSC ---- MT ---- discharge 4
5	TSC ---- MT ---- discharge 5
6	TSC ---- No MT ---- discharge 1
7	TSC ---- No MT ---- discharge 2
8	TSC ---- No MT ---- discharge 3
9	TSC ---- No MT ---- discharge 4
10	TSC ---- No MT ---- discharge 5
11	TSC ---- MT ---- transfer MCO ---- discharge 1
12	TSC ---- MT ---- transfer MCO ---- discharge 2
13	TSC ---- MT ---- transfer MCO ---- discharge 3
14	TSC ---- MT ---- transfer MCO ---- discharge 4
15	TSC ---- MT ---- transfer MCO ---- discharge 5
16	TSC ---- No MT ---- transfer MCO ---- discharge 1
17	TSC ---- No MT ---- transfer MCO ---- discharge 2
18	TSC ---- No MT ---- transfer MCO ---- discharge 3
19	TSC ---- No MT ---- transfer MCO ---- discharge 4
20	TSC ---- No MT ---- transfer MCO ---- discharge 5
21	TSC ---- Other centers
22	Other SU ---- No MT ---- discharge 1
23	Other SU ---- No MT ---- discharge 2
24	Other SU ---- No MT ---- discharge 3
25	Other SU ---- No MT ---- discharge 4
26	Other SU ---- No MT ---- discharge 5
27	Other SU ---- transfer PCF ---- discharge 1
28	Other SU ---- transfer PCF ---- discharge 2
29	Other SU ---- transfer PCF ---- discharge 3
30	Other SU ---- transfer PCF ---- discharge 4
31	Other SU ---- transfer PCF ---- discharge 5
32	Other SU ---- transfer TSC ---- MT ---- discharge 1
33	Other SU ---- transfer TSC ---- MT ---- discharge 2
34	Other SU ---- transfer TSC ---- MT ---- discharge 3
35	Other SU ---- transfer TSC ---- MT ---- discharge 4
36	Other SU ---- transfer TSC ---- MT ---- discharge 5
37	Other SU ---- transfer TSC ---- MT ---- transfer MCO ---- discharge 1
38	Other SU ---- transfer TSC ---- MT ---- transfer MCO ---- discharge 2
39	Other SU ---- transfer TSC ---- MT ---- transfer MCO ---- discharge 3
40	Other SU ---- transfer TSC ---- MT ---- transfer MCO ---- discharge 4
41	Other SU ---- transfer TSC ---- MT ---- transfer MCO ---- discharge 5
42	Other SU ---- transfer TSC ---- No MT ---- transfer MCO (or not) ---- discharge 1
43	Other SU ---- transfer TSC ---- No MT ---- transfer MCO (or not) ---- discharge 2

Number	Therapeutic pathways
44	Other SU ---- transfer TSC ---- No MT ---- transfer MCO (or not) ---- discharge 3
45	Other SU ---- transfer TSC ---- No MT ---- transfer MCO (or not) ---- discharge 4
46	Other SU ---- transfer TSC ---- No MT ---- transfer MCO (or not) ---- discharge 5
47	Other SU ---- Other centers
48	PCF ---- No MT ---- discharge 1
49	PCF ---- No MT ---- discharge 2
50	PCF ---- No MT ---- discharge 3
51	PCF ---- No MT ---- discharge 4
52	PCF ---- No MT ---- discharge 5
53	PCF ---- transfer Other SU ---- discharge 1
54	PCF ---- transfer Other SU ---- discharge 2
55	PCF ---- transfer Other SU ---- discharge 3
56	PCF ---- transfer Other SU ---- discharge 4
57	PCF ---- transfer Other SU ---- discharge 5
58	PCF ---- transfer TSC ---- No MT ---- discharge 1
59	PCF ---- transfer TSC ---- No MT ---- discharge 2
60	PCF ---- transfer TSC ---- No MT ---- discharge 3
61	PCF ---- transfer TSC ---- No MT ---- discharge 4
62	PCF ---- transfer TSC ---- No MT ---- discharge 5
63	PCF ---- transfer TSC ---- MT ---- discharge 1
64	PCF ---- transfer TSC ---- MT ---- discharge 2
65	PCF ---- transfer TSC ---- MT ---- discharge 3
66	PCF ---- transfer TSC ---- MT ---- discharge 4
67	PCF ---- transfer TSC ---- MT ---- discharge 5
68	PCF ---- transfer TSC ---- No MT ---- transfer MCO ---- discharge 1
69	PCF ---- transfer TSC ---- No MT ---- transfer MCO ---- discharge 2
70	PCF ---- transfer TSC ---- No MT ---- transfer MCO ---- discharge 3
71	PCF ---- transfer TSC ---- No MT ---- transfer MCO ---- discharge 4
72	PCF ---- transfer TSC ---- No MT ---- transfer MCO ---- discharge 5
73	PCF ---- transfer TSC ---- MT ---- transfer MCO ---- discharge 1
74	PCF ---- transfer TSC ---- MT ---- transfer MCO ---- discharge 2
75	PCF ---- transfer TSC ---- MT ---- transfer MCO ---- discharge 3
76	PCF ---- transfer TSC ---- MT ---- transfer MCO ---- discharge 4
77	PCF ---- transfer TSC ---- MT ---- transfer MCO ---- discharge 5
78	PCF ---- Other centers

SU: stroke unit, TSC: thrombectomy-capable stroke center, MT: mechanical thrombectomy, PCF: primary care facility, discharge 1: home, discharge 2: SSR, discharge 3: HAD, discharge 4: USCD, PSY, USLD, discharge 5: death.
